# Gelatine Forming Micro-Organisms

**Published:** 1886-10

**Authors:** G. V. Black

**Affiliations:** Jacksonville, Ill.


### GELATINE FORMING MICRO-ORGANISMS.

BY G. V. BLACK, M. D., D. D. S., JACKSONVILLE, ILL.

I have succeeded in isolating several species of gelatine forming oral cocci, which are very much alike, however, except as to rapidity of growth. The one in which I have been most interested is a wonderfully fickle plant, so much so that I have had great difficulty to maintain continuous growth of it. It is often impossible to replant or transfer it after the lapse of twenty-four hours. It seems necessary to do this twice or three times daily to keep it going. A tube planted with a colony picked out of a gelatine plate becomes almost as white as milk within three hours, and within fifteen to twenty-four hours the entire contents of the tube (peptonized broth with 2% of sugar,) is gelatinized so perfectly that upon inverting it there will, at most, only a drop of clear fluid appear. This gelatine is for the most part built up from the bottom, or from the sides of the tube. There is nothing like a film on the top, at any time. It seems to grow best at a temperature above 100° F. and my observations thus far go to show that it is essentially the organism of *sordes in fevers.* The tubes have a faint yellowish cast, and in case all the fluid is not solidified, that which remains is as clear as crystal, and markedly acid in reaction. In gelatine tubes it grows in the form of vesicles, the contents of which are lighter in color and more transparent than the gelatine about them, and on gelatine plates its colony looks more like the pits left in the skin by small-pox, than anything else with which I can compare it. They appear sunken, very transparent, and a shade lighter than the surrounding portions. They do not grow large enough to be seen by the naked eye, and if not taken off early refuse to grow in any of the media that I have tried. In most persons whom I have examined, this coccus is found far back on the dorsum of the tongue, and occasionally I find it scattered generally through the mouth. It is a Streptococcus in form of growth, but rarely forms chains of more than five or six cocci. It does not grow in pairs, or even numbers, as the other streptococci, odd numbers being seen in the chain about as often as even numbers. Yet very many diplococcus forms appear from the division of single cells.

Another form grows very slowly, requiring a week to develop. The tubes have a decided bluish cast, and the amount of gelatine found is small. In this the chains of cocci are generally longer and more regularly formed.

This A. M. I made a plant which I have held at 103° F. Now, at 4 P. M., I have four generations from it, each plant being taken after the tubes were decidedly milky. The original tube is now half full of gelatine. This will give some idea of what this coccus may do in gumming up the teeth, tongue, etc., when fever occurs, which with its high temperature perfects the conditions for its growth. It also gives a reason for the non-appearance of sordes in some cases of fever. *The coccus is not present in every mouth.* I should say that gelatine is not quite the proper word to use in this connection. The substance does not melt or soften at high temperatures, and in many respects differs from what we know as gelatine, but it is more like that, as it appears in the tubes, than anything else with which I am acquainted.

